# Complete Genome Characterization of the Porcine Deltacoronavirus HKD/JPN/2016, Isolated in Japan, 2016

**DOI:** 10.1128/genomeA.00795-17

**Published:** 2017-08-24

**Authors:** Tohru Suzuki, Jun Hayakawa, Seiichi Ohashi

**Affiliations:** a Viral Disease and Epidemiology Research Division, National Institute of Animal Health, NARO, Tsukuba, Ibaraki, Japan; b Oshima Livestock Hygiene Service Center, Hakodate, Hokkaido, Japan

## Abstract

In 2016, an outbreak of diarrhea with high mortality in piglets occurred on a swine farm in Hokkaido prefecture, Japan. The causative porcine deltacoronavirus HDK/JPN/2016 was isolated from intestinal samples of the dead piglets on LLC-PK1 cells. The complete genome of HKD/JPN/2016 was sequenced and analyzed by next-generation sequencing technology.

## GENOME ANNOUNCEMENT

Recently, the traditional three classifications in the *Coronavirinae* subfamily were replaced by adding another novel genus, *Deltacoronavirus*, which is found in diverse host species, including some mammalian and avian species (1–3). In a large-scale surveillance study done in Hong Kong, additional deltacoronaviruses were identified in many kinds of avian species and swine (4). Then, porcine deltacoronavirus (PDCoV) was first detected from pigs with diarrhea in Ohio, USA, in 2014, and has been frequently identified in other states (5–8). PDCoV has also been reported in some Asian countries, including South Korea, China, Thailand, and Laos (9–12).

In Japan, PDCoVs have often been detected in diarrheic domestic pigs at swine farms in multiple prefectures since 2014. In September 2016, a unique case of diarrhea caused by PDCoV occurred in one commercial swine farm (1,300 sows) in Hokkaido prefecture. Briefly, approximately 280 piglets (mainly under 3 days old) and some sows showed acute, watery diarrhea, and over 50 of the piglets (approximately 20% of mortality) died within 1 week. The surviving piglets and sows recovered from the diarrhea after 2 weeks. The causative PDCoV strain, HKD/JPN/2016, was isolated from LLC-PK1 (ECACC, 86121112) cell lines from intestinal homogenates of the dead piglets.

RNA from the HKD/JPN/2016 isolate was reverse transcribed and PCR amplified by using originally designed primer sets against the PDCoV genome. The amplified genomes were sequenced using next-generation sequencing technology on an Ion PGM platform (Thermo Fisher Scientific, Carlsbad, CA, USA) according to the manufacturer’s instructions. The data were assembled using Torrent Suite version 5.0 based on known complete genomes of PDCoV strains obtained in the United States, South Korea, China, Hong Kong, Thailand, and Laos (4–12).

The nearly complete genomic sequence was found to be 25,359 nucleotides (nt) in length, including partial unidentified regions at both the 5′ and 3′ terminals. There were also no changes in the length of each gene, other than a 3-nt deletion in the *ORF1a/1b* gene of the U.S. and South Korean PDCoV strains. Comparative sequence analysis of the complete genome of HKD/JPN/2016 showed 97.0 to 99.6% nt diversity to the genomes of the 49 reference strains from the United States, South Korea, China, Hong Kong, Thailand, Laos, and Vietnam available in GenBank; the highest nt identities were to the South Korean PDCoV strain, KNU14-04/KOR/2014. A phylogenetic dendrogram using complete genomes from 50 PDCoV strains indicated that the HKD/JPN/2016 isolate was closely related with PDCoV strains from the United States and Korea detected in recent years; moreover, the isolate was divided into a branch separate from the KNU14-04/KOR/2014 strain. These findings suggest that the HKD/JPN/2016 isolate was naturally derived from origins as common as the widespread PDCoVs detected in the United States and South Korea since late 2013. In a previous study, the Thai PDCoV, which had nt deletions and/or insertions in the genomic sequence compared to those of other PDCoVs, exhibited high mortality (about 20%) in piglets at a commercial swine farm (10). These data suggest the possibility that the unique genomic sequence of HKD/JPN/2016 might be associated with the death of diarrheic piglets. A continuous molecular characterization of novel PDCoV is essential for monitoring the genomic dynamics of PDCoV worldwide.

### Accession number(s).

The complete genome of the isolate HKD/JPN/2016 has been deposited in GenBank under the accession no. LC260045.
